# Loss of the Homeodomain Transcription Factor Prep1 Perturbs Adult Hematopoiesis in the Bone Marrow

**DOI:** 10.1371/journal.pone.0136107

**Published:** 2015-08-18

**Authors:** Kentaro Yoshioka, Akihisa Oda, Chihiro Notsu, Takafumi Ohtsuka, Yasuhiro Kawai, Sadafumi Suzuki, Takuro Nakamura, Yo Mabuchi, Yumi Matsuzaki, Ryo Goitsuka

**Affiliations:** 1 Division of Development and Aging, Research Institute for Biomedical Sciences, Tokyo University of Science, Noda, Chiba, Japan; 2 Department of Physiology, Keio University School of Medicine, Shinjuku-ku, Tokyo, Japan; 3 Division of Carcinogenesis, The Cancer Institute, Japanese Foundation for Cancer Research, Koto-ku, Tokyo, Japan; 4 Department of Biochemistry and Biophysics, Graduate School of Health Care Sciences, Tokyo Medical and Dental University, Bunkyo-ku, Tokyo, Japan; 5 Department of Cancer Biology, Faculty of Medicine, Shimane University, Izumo-shi, Shimane, Japan; St. Vincent's Institute, AUSTRALIA

## Abstract

Prep1, a TALE-family homeodomain transcription factor, has been demonstrated to play a critical role in embryonic hematopoiesis, as its insufficiency caused late embryonic lethality associated with defective hematopoiesis and angiogenesis. In the present study, we generated hematopoietic- and endothelial cell-specific Prep1-deficient mice and demonstrated that expression of Prep1 in the hematopoietic cell compartment is not essential for either embryonic or adult hematopoiesis, although its absence causes significant hematopoietic abnormalities in the adult bone marrow. Loss of Prep1 promotes cell cycling of hematopoietic stem/progenitor cells (HSPC), leading to the expansion of the HSPC pool. Prep1 deficiency also results in the accumulation of lineage-committed progenitors, increased monocyte/macrophage differentiation and arrested erythroid maturation. Maturation of T cells and B cells is also perturbed in Prep-deficient mice. These findings provide novel insight into the pleiotropic roles of Prep1 in adult hematopoiesis that were unrecognized in previous studies using germline *Prep1* hypomorphic mice.

## Introduction

Many diverse functions have been described for the three-amino-acid-loop-extension (TALE) class of homeodomain transcription factors during embryonic and postnatal development in vertebrates [1]. These transcription factors, which include the Meis, Prep and Pbx families, share a conserved atypical homeodomain containing a three-amino acid loop extension between the first two α-helices, through which they can bind to the target DNA as well as interact with Hox proteins [2]. In addition, Prep and Meis family members have two additional conserved domains in their N-terminal region, the MEIS-A and MEIS-B domains (also termed HM1 and HM2), which function as an interface for hetero-dimerization with Pbx family members [3–6], promoting nuclear translocation and also affecting DNA-binding choice by the Pbx proteins. Thus, the MEIS-A/B domain of Meis/Prep family proteins is a key regulatory domain that can mediate both positive and negative effects on their biological functions.

The Prep family consists of Prep1 and Prep2 in mice and humans [7–10], and Prep1 is relatively ubiquitously expressed in most tissues [8, 11]. As with other TALE homeodomain proteins, targeted gene disruption in mice has advanced our understanding of the function of Prep1 in physiological and pathological conditions, such as in hematopoietic cell development, T cell development in the thymus, and in the suppression of tumor formation [12–15]. Several mouse strains harboring different *Prep1*-mutant alleles have been established; however, the phenotypes observed in each mouse strain are variable, probably depending on the level of the remaining trace amounts of intact and/or mutant Prep1 proteins and the genetic background of the mice. For instance, *Prep1*-deficient embryos that have been reported to express no intact Prep1 protein die at E7.5 shortly after implantation, with massive apoptosis and proliferation defects [16]. In contrast, *Prep1* hypomorphs, in which RNA expression is reduced to about 2% of wild-type levels due to a retroviral insertion in the first intron of *Prep1* gene, mostly die at E17.5 or later, showing defects in hematopoiesis, eye development and angiogenesis [13, 17]. In addition, a small number of *Prep1* hypomorphic mice escaped embryonic lethality and were born and survived till the adulthood, although they displayed multiple anomalies in certain tissues [18–20]. Recently, two laboratories generated mouse lines carrying different conditional *Prep1* alleles, one with *lox*P sites flanking exon 8 encoding the N-terminal part of the homeodomain [21] and the other with *lox*P sites flanking exons 6 and 7, which encode an intervening region between the MEIS-B domain and the homeodomain [15]. Both of these alleles, however, still retain the potential to generate truncated Prep1 proteins lacking the homeodomain but retaining the MEIS-A/B domain, which may affect the interaction of other Meis/Prep proteins with Pbx family members. Indeed, Carbe *et al*. have demonstrated the expression of such a truncated protein from the mutant *Prep1* allele by Western blotting [21]. Furthermore, although a *trans*-heterozygote Prep1 mutant harboring one hypomorphic allele and one null allele had lens induction defects [22], the conditional deletion of exon 8 of the *Prep1* gene at the placodal phase of lens induction failed to cause any lens defects [21], suggesting a potential function of the homeodomain-less Prep1 protein other than direct binding to its target genes. In this regard, homeodomain-less isoforms have been described for TALE class homeodomain transcription factors. Alternative splicing of the *Drosophila* orthologue of *Meis1*, *Homothorax*, generates a homeodomain-less isoform that only contains MEIS-A/B domains, and this homeodomain-less protein is sufficient to carry out most Homothorax functions [23], supporting the existence of homeodomain-independent functions of the MEIS-A/B domain. In mammals, Meis and Prep isoforms lacking a homedomain have also been identified in certain tissues and cells [24] and, in the case of Meis2, it acts as a dominant-negative regulator by competing with homeodomain-containing isoforms for DNA binding [25]. Thus, truncated Prep1 proteins retaining a MEIS-A/B domain could potentially act as either negative and positive regulators, the outcome depending on the composition of Meis as well as Pbx members inside a given cell at distinct differentiation stages. Therefore, to clearly understand the pleiotropic functions of Prep1 in various biological processes, the phenotypes of cell type-specific *Prep1* null mutations need to be clearly defined.

To circumvent the problem of potential retention of functional mutant Prep1 proteins and the confounding compensatory mechanisms arising due to germline *Prep1* insufficiency in a given cell type, we generated mice carrying a new conditional allele, with *lox*P-flanked exon 3 of the *Prep1* gene that acts as a null allele upon Cre-mediated recombination. We then examined the cell autonomous functions of Prep1 in hematopoiesis by crossing this allele with a hematopoietic- and endothelial-specific *Tie2-Cre* mouse strain.

## Results

### Generation of hematopoietic/endothelial cell-specific Prep1-deficient mice

To examine the role of Prep1 in the adult mouse hematopoietic system, we generated mice harboring conditional *Prep1* (*Prep* ^*fl*^) alleles, in which exon 3 of the *Prep1* gene encoding the N-terminal part of the MEIS-A domain is floxed by *lox*P sites (S1 Fig). The *Prep1* ^fl/fl^ mice were mated with *Tie2-Cre* mice, in which Cre is specifically expressed in endothelial cells and hematopoietic stem cells [26], to generate *Tie2-Cre;Prep1* ^*fl/fl*^ mice (designated as CKO mice). PCR analysis of the resulting CKO mice showed specific and complete deletion of exon 3 of the *Prep1* gene in the bone marrow (BM) (S1 Fig). Removal of exon 3 is predicted to induce a frameshift mutation, leading to the incorporation of 11 unrelated amino acids before encountering a termination codon within the exon 4-encoded sequence. Consistently, a transcript from the mutant allele lacking exon 3 sequence was detected in CD45^+^ hematopoietic cells from the BM of CKO mice by RT-PCR using primers corresponding to DNA sequence unique to exon 2 and exon 6 (S1 Fig), and confirmed by subsequent cDNA sequencing (S1 Fig). Prep1 mutant proteins were also undetectable in the hematopoietic cells from CKO BM by Western blotting using an antibody directed against the N-terminal portion of Prep1 (S1 Fig). Thus, these findings indicate that any hematopoietic phenotypes observed in our CKO mice are the result of the Prep1 null-phenotype.

### Expression of Prep1 in hematopoietic and endothelial cells is dispensable for embryonic hematopoiesis in the fetal liver

The CKO mice were born at predicted Mendelian frequencies and appeared normal at birth (data not shown), indicative of proper embryonic development in the absence of Prep1 in hematopoietic and endothelial cells. This result was totally unexpected, since previous studies indicated that the embryonic lethality resulting from Prep1 deficiency was partly due to hematopoietic and angiogenic defects [13, 17]. We thus examined the hematopoietic compartment in the fetal liver of E14.5 CKO embryos. Total numbers of fetal liver cells in CKO embryos were consistently somewhat higher than those of control embryos, and the numbers of cells in the immature hematopoietic compartments, including LSK (Lin^-^ Sca-1^+^c-Kit^+^) enriched in hematopoietic stem/progenitor cells (HSPCs), common lymphoid progenitor (CLP; Lin^-^IL-7Rα^+^Sca-1^int^c-Kit^+^), common myeloid progenitor (CMP; Lin^-^Sca-1^-^c-Kit^+^FcγRII/III^int^ CD34^high^), granulocyte/monocyte progenitor (GMP: Lin^-^Sca-1^-^c-Kit^+^FcγRII/III^high^ CD34^high^) and megakaryocyte/erythroid progenitor (MEP; Lin^-^Sca-1^-^c-Kit^+^FcγRII/III^-^ CD34^low^) in CKO fetal livers were comparable to those in control fetal livers (S2 Fig). Furthermore, the differentiation of B cells, monocytes, granulocytes and erythrocytes appeared to be intact in CKO fetal livers (S2 Fig). Thus, these findings indicate that Prep1 expressed in the hematopoietic and endothelial cell compartments is dispensable for embryonic hematopoiesis in the fetal liver.

#### Effects of Prep1 deficiency on adult HSPC and lineage-committed progenitor compartments

**Fig 1:**
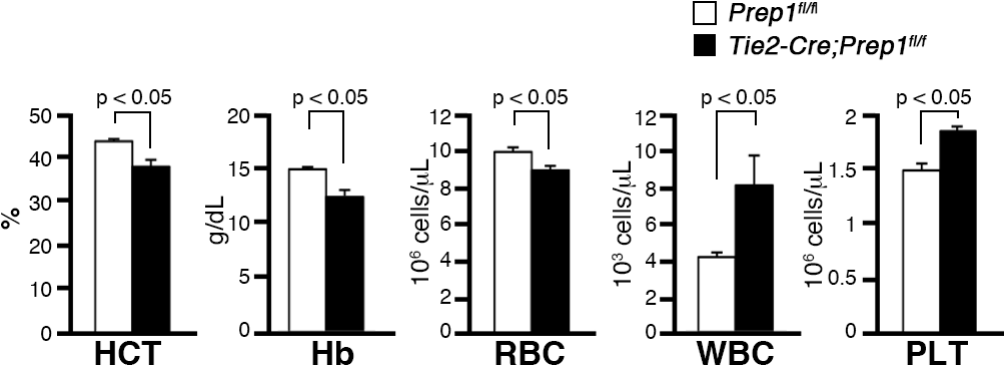
Hematological indices of *Tie2-Cre; Prep1* ^*fl/fl*^ mice. *Tie2-Cre; Prep1* ^fl/fl^ mice (solid bars) and *Prep1* ^fl/fl^ littermate controls (open bars), n = 4 for each group were analyzed 8 weeks after birth. Bar graphs indicate mean and SD; HCT, hematocrit; Hb, hemoglobin; RBC, red blood cell count; WBC, white blood cell count; PLT, platelet count.

**Fig 2:**
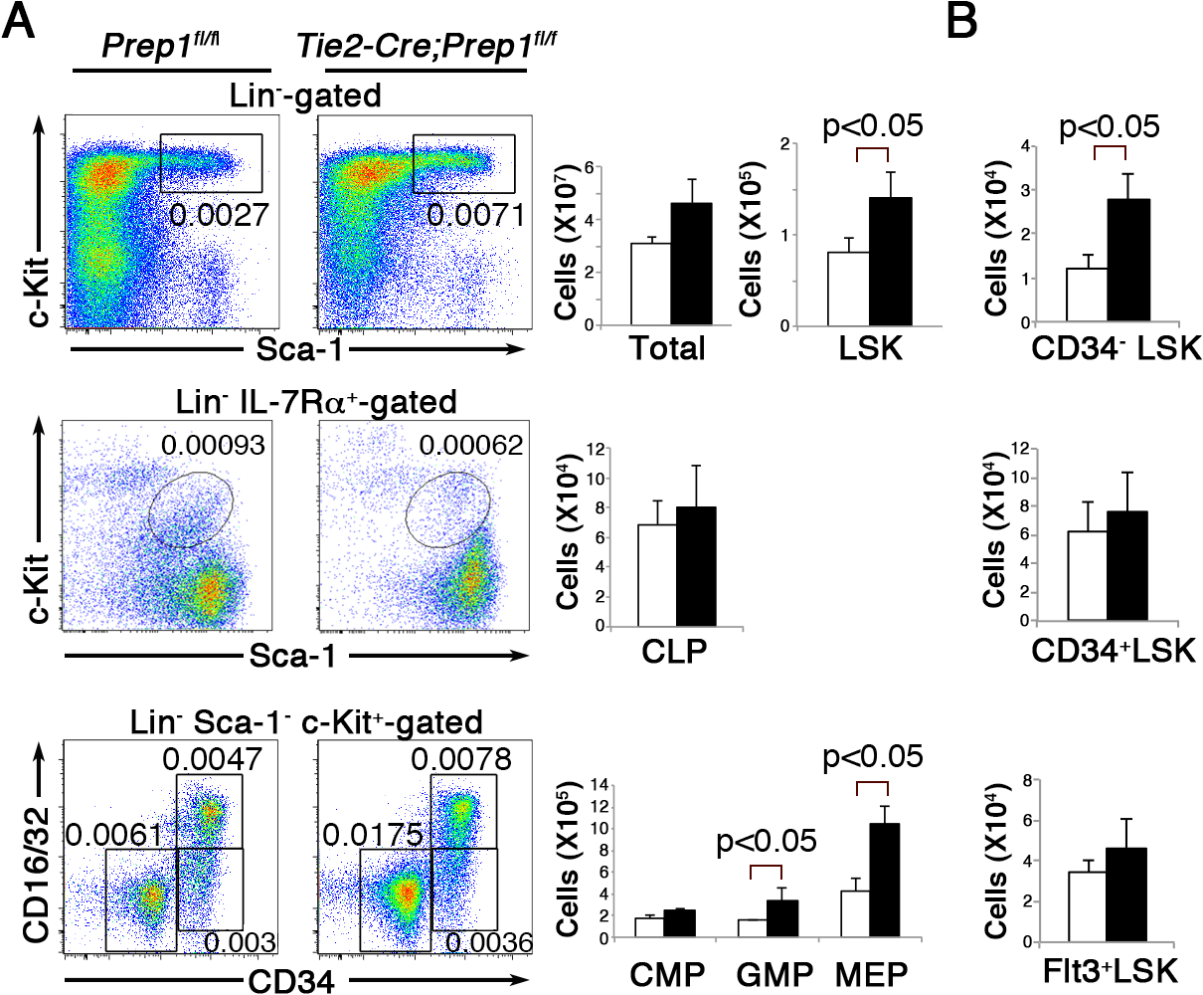
Loss of Prep1 leads to an expansion of hematopoietic stem/progenitor cell compartments in the bone marrow. (**A**) Representative flow cytometric profiles of hematopoietic progenitor cell populations (LSK; top panel, CLP; middle panel, CMP, GMP and MEP; bottom panel) from the BM of *Tie2-Cre; Prep*1^*fl/fl*^ mice and *Prep1* ^*flfl*^ littermate controls. Gates used to identify the progenitor populations are indicated. Numbers indicate the percentage of gated cells in total BM mononuclear cells. Bar graphs on the right represent absolute numbers of the indicated cell populations per two femurs in *Tie2-Cre; Prep*1^*fl/fl*^ (solid bars) and control *Prep1* ^*flfl*^ littermate (open bars) mice (mean and SD; n = 4). (**B**) Bar graphs depict the absolute numbers of the indicated cell populations per two femurs in *Tie2-Cre; Prep*1^*fl/fl*^ (solid bars) and control *Prep1* ^*flfl*^ littermate (open bars) mice (mean and SD; n = 4).

To investigate the effect of Prep1 loss in the hematopoietic and endothelial cell compartments on adult hematopoiesis, we first analyzed the peripheral blood of 8-week-old CKO and littermate control mice. In CKO mice, we found decreases in red blood cell number, hematocrit values and hemoglobin concentration and an increase in the number of white blood cells and platelets (Fig 1), indicating hematopoietic abnormalities affecting nearly all lineages. We therefore examined the primitive hematopoietic cell compartments in the BM. As observed in the fetal liver, total numbers of BM cells were slightly, but not significantly, increased in CKO mice as compared to those in control mice (Fig 2A). At the stem/progenitor level, the numbers of LSK cells enriched in HSPCs were significantly elevated in CKO mice (Fig 2A). Although total numbers of CLP and CMP cells in CKO mice were comparable to those in control mice, the cell numbers at the subsequent developmental stages, including GMP and MEP, were significantly elevated in CKO mice (Fig 2A). When we examined the LSK compartment in more detail by staining for CD34 or Flt3 expression, the total number of the most immature CD34^-^ LSK cells was significantly elevated, while the mature CD34^+^LSK and Flt3^+^LSK cell populations were slightly but not significantly expanded in CKO mice compared to controls (Fig 2B). These results suggest that Prep1 expressed in the hematopoietic and endothelial cell compartments is not required for adult hematopoiesis in the BM, but its absence causes an increase in the immature HSPC pool size.

### Prep1 deficiency compromises cell cycle status of the HSPC compartment

**Fig 3:**
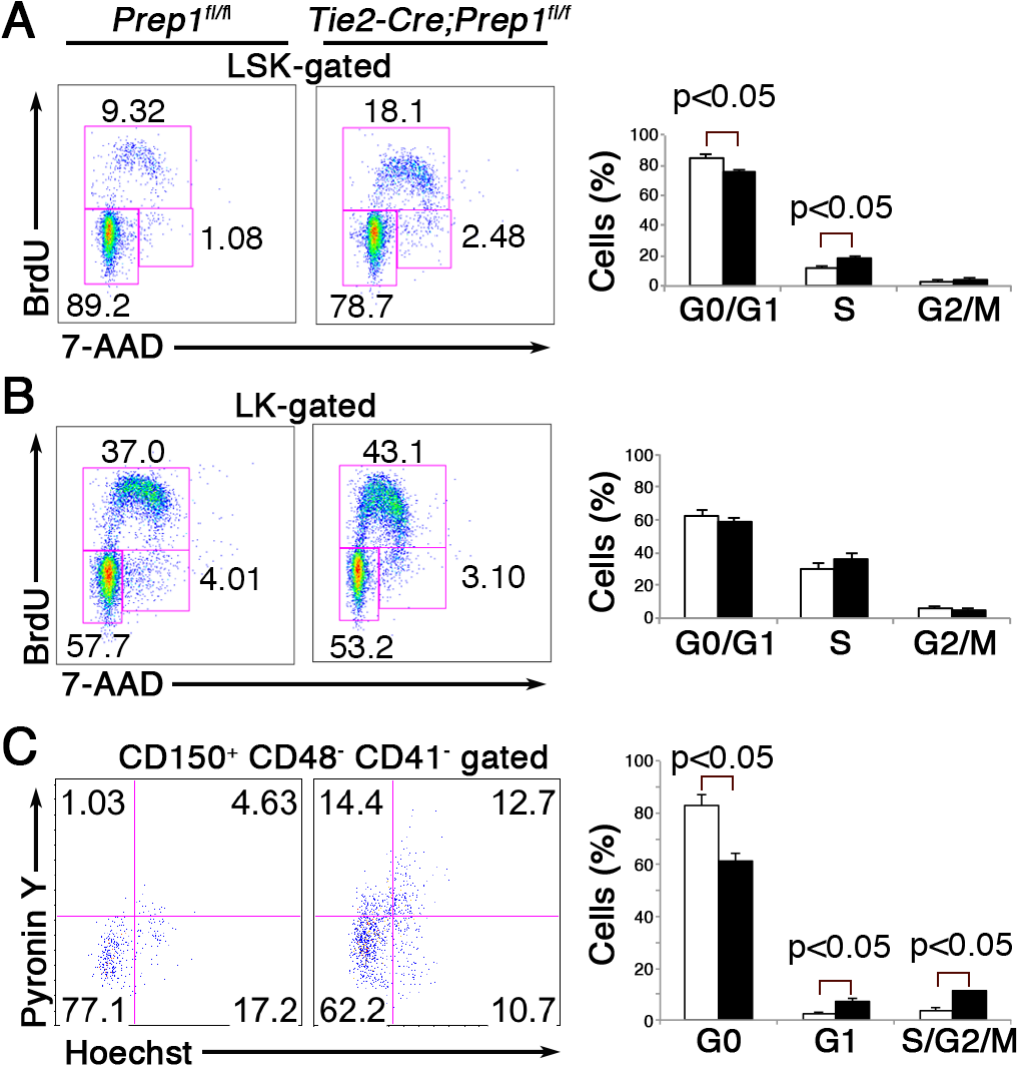
Loss of Prep1 enhances cell cycling in the HSPC compartment. (**A**) Representative flow cytometric profiles showing BrdU incorporation and 7-AAD staining of LSK cells from *Tie2-Cre; Prep*1^*fl/fl*^ and control mice. Numbers indicate percentage of gated cells at the different cell cycle stages. Bar graphs shown on the right depict the percentages of cells in G0/G1-, S- and G2/M-phase of the cell cycle in the LSK cell population from *Tie2-Cre; Prep*1^*fl/fl*^ (solid bars) and control *Prep1* ^*flfl*^ littermate (open bars) mice (mean and SD; n = 3). (**B**) Representative flow cytometric profiles showing BrdU incorporation and 7-AAD staining of Lin^-^ Sca1^-^ c-Kit^+^ (LK) cells, mainly containing myeloid progenitors, from *Tie2-Cre; Prep*1^*fl/fl*^ and control mice. Bar graphs on the right depict the percentages of cells in G0/G1-, S- and G2/M-phase of the cell cycle in the LK cell population from *Tie2-Cre; Prep*1^*fl/fl*^ (solid bars) and control *Prep1* ^*flfl*^ littermate (open bars) mice (mean and SD; n = 3). (C) Representative flow cytometric profiles showing PyroninY and Hoechst 33342 staining of CD150^+^ CD48^-^ CD41^-^ SP cells from *Tie2-Cre; Prep*1^*fl/fl*^ and control mice. Bar graphs on the right depict the percentages of cells in G0 -, G1- and S/G2/M-phase of the cell cycle in the indicated cell population from *Tie2-Cre; Prep*1^*fl/fl*^ (solid bars) and control *Prep1* ^*flfl*^ littermate (open bars) mice (mean and SD; n = 3).

To understand the mechanisms of the increase in the HSPC pool size upon Prep1 loss, we examined the cell proliferation status of HSPCs by using BrdU incorporation. As shown in Fig 3A, the number of cells in S phase was significantly increased in CKO LSK cells, and this was accompanied by a significant reduction in the G0/G1 fraction, compared to control LSK cells. In contrast, the cell cycle status of the LK cell population, which mainly contains more differentiated progenitors such as CMP, GMP and MEP, was unaltered in CKO mice (Fig 3B). Furthermore, cell cycle analysis of the CD150^+^ CD48^-^ CD41^-^ “side population” (SP) cells, representing HSPCs as immature LSK cells [27], from CKO mice revealed a significant decrease in G0 and an increase in the G1 and S/G2/M phases (Fig 3C). Therefore, the increased frequency of cycling HSPCs, but not of more differentiated progenitor cells, due to the loss of Prep1 in the hematopoietic and endothelial cell compartments appears to cause the expansion of the HSPC pool size.

### Impact of Prep1 deficiency on the differentiation of erythroid/megakaryocytic- and myeloid-lineage cells

**Fig 4:**
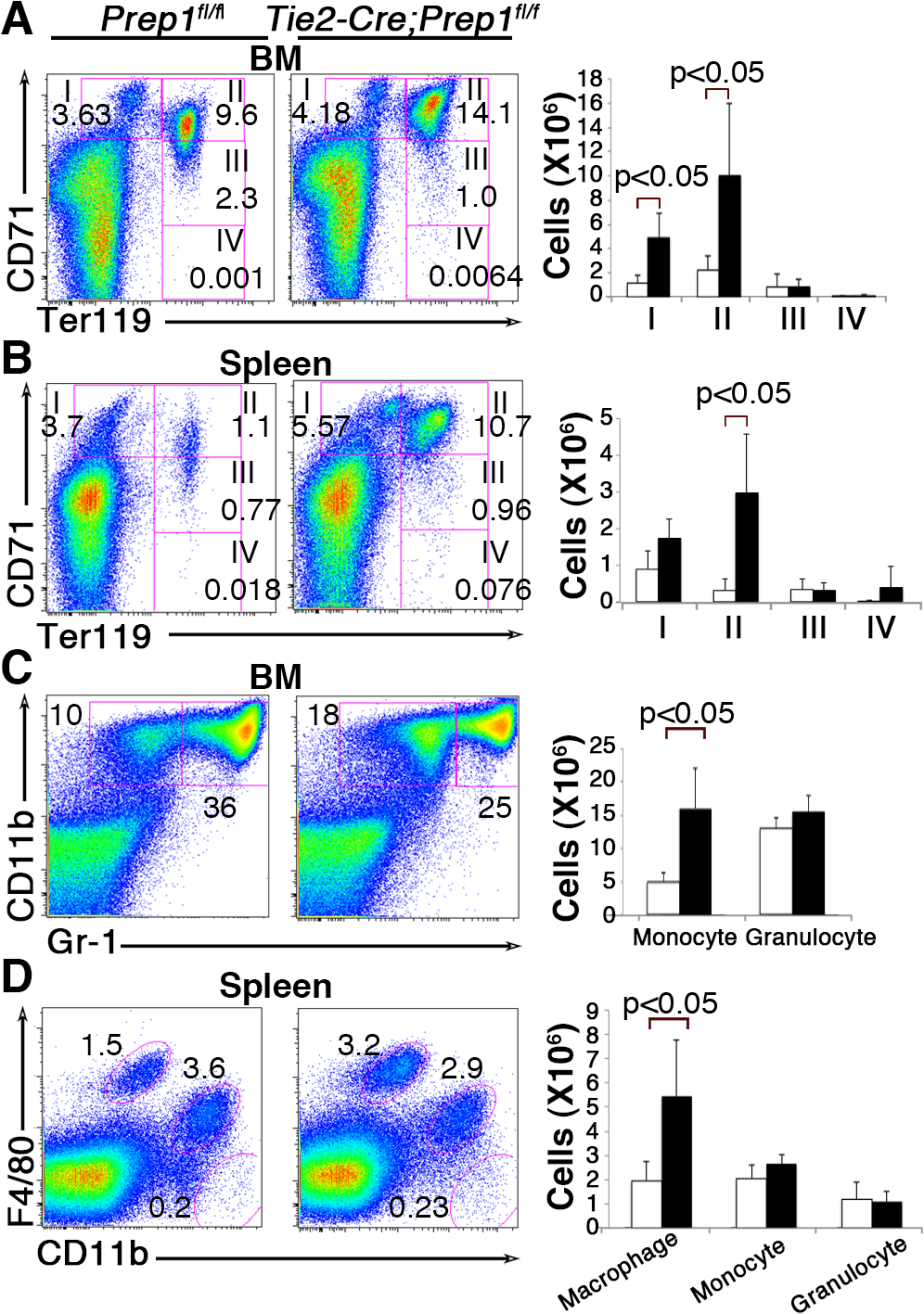
Impact of Prep1 loss on the differentiation of erythroid- and myeloid-lineage cells. (**A**, **B**) Representative flow cytometric profiles of erythroid-lineage cells in the BM (**A**) and spleen (**B**). Numbers indicate percentage of gated cells among the total cells analyzed. Roman numerals indicate the developmentally defined subpopulations of erythroblasts: I, proerythroblasts; II, basophilic erythroblast; III, polychromatophilic erythroblasts; IV, orthochromatophilic erythroblasts. Bar graphs on the right depict absolute numbers of the indicated cell populations in the BM of two femurs and the whole spleen in *Tie2-Cre; Prep*1^*fl/fl*^ (solid bars) and control *Prep1* ^*flfl*^ littermate (open bars) mice (mean and SD; n = 4). (**C**, **D**) Representative flow cytometric profiles of myeloid-lineage cells in the BM (**C**) and spleen (**D**). Numbers indicate percentage of gated cells among the total cells analyzed; granulocytes (Gr-1^+^ CD11b^+^) and monocytes (Gr-1^-^ CD11b^+^) in **C**, granulocytes (F4/80^-^ CD11b^high^), monocytes (F4/80^dull^ CD11b^dull^) and macrophages (F4/80^high^ CD11b^low^). Bar graphs on the right depict absolute numbers of the indicated cell populations in the BM of two femurs and the whole spleen in *Tie2-Cre; Prep*1^*fl/fl*^ (solid bars) and control *Prep1* ^*flfl*^ littermate (open bars) mice (mean and SD; n = 4).

We next examined the effect of Prep1 deficiency on the mature cell compartments in the BM and spleen. As shown in Fig 4A, the analysis of erythroid/megakaryocytic-lineage differentiation in the BM revealed ineffective erythropoiesis in CKO mice characterized by a significant increase in the frequency and number of proerythroblasts (I; Ter119^low^ CD71^high^) and early basophilic erythroblasts (II; Ter119^high^ CD71^high^). In contrast, megakaryocytic-lineage cell differentiation in the BM was intact in CKO mice (S3 Fig). In the mouse, inefficient erythropoiesis in the BM can be compensated by stress erythropoiesis in the spleen [28, 29]. As expected, the absolute number of early basophilic erythroblasts in the spleen was ~10-fold increased in CKO mice as compared to control mice (Fig 4B), suggesting a partial compensation by expansion of the early erythroid compartment in the spleen.

In contrast to the partially arrested erythroid differentiation, the frequencies and the total numbers of monocytes (Gr-1^-^ CD11b^+^), but not granulocytes (Gr-1^+^CD11b^+^), were significantly elevated in the BM of CKO mice, as compared to controls (Fig 4C **)**. When we examined monocyte/macrophage-lineage cells in the spleen [30], the number of F4/80^high^ CD11b^-^ macrophages was also significantly higher in CKO mice than in controls (Fig 4D), suggesting a biased impact of Prep1 deficiency on differentiation of the monocyte/macrophage-lineage rather than the granulocytic-lineage. Taken together, these findings indicate a potential role of Prep1 in erythroid cell maturation as well as in monocyte/macrophage differentiation.

### Impact of Prep1 deficiency on the differentiation of lymphoid-lineage cells

**Fig 5:**
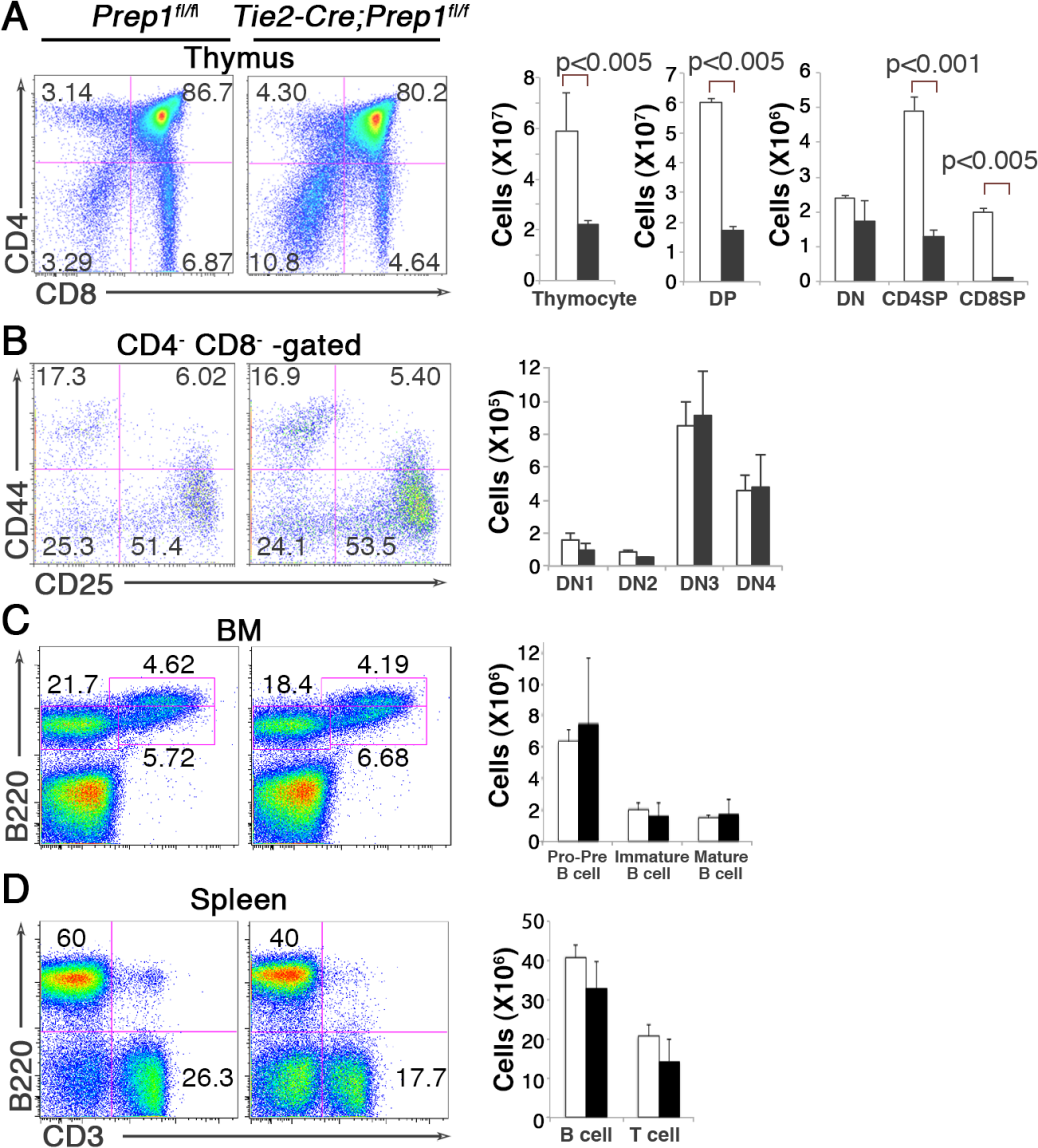
Impact of Prep1 loss on the differentiation of lymphoid-lineage cells. (**A, B**) Representative flow cytometric profiles of T cell progenitor populations stained with anti-CD4 and anti-CD8 (**A**) and the CD4^−^CD8^−^ cell population stained with anti-CD44 and anti-CD25 (**B**) in the thymus from *Tie2-Cre; Prep*1^*fl/fl*^ and control *Prep*1^*fl/fl*^ mice. Numbers in each quadrant indicate percentage of total cells analyzed. Bar graphs on the right depict absolute numbers of CD4^−^CD8^−^ (DN), CD4^+^CD8^+^ (DP), CD4^+^CD8^−^ (CD4 SP), CD4^−^CD8^+^ (CD8 SP), CD44^+^ CD25^-^ (DN1), CD44^+^ CD25^+^ (DN2), CD44^-^ CD25^+^ (DN3), and CD44^-^ CD25^-^ (DN4) cell populations in *Tie2-Cre; Prep*1^*fl/fl*^ (solid bars) and control *Prep1* ^*flfl*^ littermate (open bars) mice (mean and SD; n = 4). (**C, D**) Representative flow cytometric profiles of B-lineage cells in the BM (**C**) and mature B and T cells in the spleen (**D**). Numbers indicate percentage of gated cells among the total cells analyzed; pro- and pre-B cells (B220^low^ IgM^-^), immature B cells (B220^low^ IgM^+^), mature B cells (B220^+^ IgM^+^) in **C**, B cells (B220^+^ CD3^-^), T cells (B220^-^ CD3^+^) and nonT/nonB cells (B220^-^ CD3^-^). Bar graphs on the right depict absolute numbers of the indicated cell populations in the BM of two femurs and the whole spleen in *Tie2-Cre; Prep*1^*fl/fl*^ (solid bars) and control *Prep1* ^*flfl*^ littermate (open bars) mice (mean and SD; n = 4).

Given that the abnormal lymphoid cell differentiation has been observed in *Prep1*-hypomorphic mice [15, 20], we also assessed differentiation of B and T cells in our Prep1-CKO mice. Similar to the *Prep1* hypomorphic mice [20], total cell numbers in the thymus were significantly reduced in CKO mice, mainly from a significant reduction in the CD4^+^ CD8^+^ double-positive (DP) thymocytes and the CD4^+^ and CD8^+^ single-positive (SP) thymocytes (Fig 5A). While the frequency of CD4^-^ CD8^-^ double-negative (DN) thymocyte population was increased, the numbers of total DN thymocytes as well as the distinct developmental stages within the DN fraction (from the DN1 to DN4 stages) were not significantly altered **(** Fig 5B), suggesting a potential role of Prep1 in the transition from the DN4 stage to the DP stage of thymocyte development and/or for the maintenance of the DP stage itself. Despite the abnormal T cell development observed in the thymus of CKO mice, the number of mature T cells in the spleen was not significantly altered (Fig 5D), which is probably due to compensation of peripheral T cell numbers by homeostatic expansion of the small population of naïve T cells exported from the CKO thymus.

In contrast to the similarity in thymocyte differentiation between the *Prep1* hypomorphic mice and our CKO mice, their B cell differentiation phenotype was somewhat different. Although *Prep1*-hypomorphic mice showed a partial arrest in early B cell differentiation [15], the total number of early B-lineage pro- and pre-B cell populations (B220^low^ IgM^-^), as well as immature and mature B cells (B220^low^ IgM^+^ and B220^high^ IgM^+^) in the BM was not significantly altered in the CKO mice (Fig 5C). In addition, the number B cells in the spleen unaltered in the CKO mice (Fig 5D).

## Discussion

Hematopoiesis is a highly orchestrated process that involves the generation of multi-lineage blood cells from a small pool of hematopoietic stem cells (HSCs) with self-renewal and multi-differentiation potentials through a successive series of increasingly lineage-restricted intermediate progenitors [31]. In the present study, we used *Tie2-Cre*-mediated hematopoietic and endothelial cell-specific deletion of the *Prep1* gene to create a complete conditional null allele in the hematopoietic and endothelial cell compartments and could demonstrate that Prep1 regulates multiple differentiation stages of hematopoietic cell development in the adult BM. Although Prep1 in the hematopoietic and endothelial cell compartments is dispensable for embryonic as well as adult hematopoiesis, its deficiency results in a number of abnormalities: HSPC expansion and partially arrested erythroid maturation in the adult BM, biased monocyte/macrophage-lineage differentiation in the adult BM as well in the spleen, and defective thymocyte differentiation in the thymus. Most of these functions of Prep1 in adult hematopoiesis are previously unrecognized, because most of our current knowledge comes from studies using germline *Prep1*-hypomorphic mice.

The observed hematopoietic phenotypes in the hematopoietic and endothelial cell-selective Prep1-CKO mice are apparently opposite those observed in the case of deficiencies in either Pbx1 or Meis1 in the hematopoietic system. *Tie2-Cre*-mediated *Pbx1* deficiency results in the increased cell cycle entry of HSCs, leading to their exhaustion, indicating an essential role of Pbx1 in the self-renewal of adult HSCs [32]. Furthermore, Pbx1 is shown to function in restraining myeloid maturation of CMPs and in maintaining lymphoid differentiation potential at the level of the CLP [33]. Meis1 has been shown to positively regulate HSC quiescence [34, 35] as well as megakaryocyte differentiation in the adult BM [36]. Similar opposing roles of Prep1 and Meis1 have been reported in *HoxA9*-induced leukemogenesis. Meis1 cooperates with *HoxA9* in leukemogenesis, as its overexpression accelerates the emergence of *HoxA9*-induced acute myeloid leukemia [37], whereas Prep1 overexpression does not accelerate *HoxA9*-induced leukemia but rather delays its onset [38]. Acceleration of *Meis-HoxA9*-induced leukemia on a *Prep1*-hypomorphic background has also been reported [39]. Therefore, in order to interpret the hematopoietic phenotype of our Prep1-CKO mice, the complex interactions of each of these three TALE family members must be taken into account, as their patterns of interaction affect gene activation and/or inhibition, as well as each other’s protein stability, all of which can lead to different biological outcomes. In this regard, recent comprehensive Chip-seq analyses of the genomic interactions of Prep1, Meis1 and Pbx1 revealed that Prep1 preferentially interacts with promoter regions of its target gene as a dimer with Pbx family members, while Meis1 has a preference for binding to intergenic as well as intragenic enhancer regions of its target genes as a trimer with Pbx and Hox [40]. Furthermore, selection of target gene specificities by Pbx1 appears to depend on its dimerization partners, Prep1 or Meis1. Thus, Prep1 and Meis1 can play opposing roles via competitive interaction with Pbx1, which leads to activation and/or inactivation of different sets of genes. In addition, it has also recently demonstrated that Prep1 competes with Meis1 for Pbx1 binding, and that Prep1 posttranscriptionally destabilizes Meis1 protein, leading to their opposing contributions to tumorigenesis [41].

Based on the above findings indicating that the constitution of Prep, Meis and Pbx family proteins expressed in a given cell type indirectly and cooperatively affects Prep1 activity, the loss of Prep1 in HSPCs might result in an exclusive association of Meis1 with Pbx1, which could enhance the activation of a subset of genes that are involved in HSC symmetric self-renewal. In this regard, Meis1 binding was observed in intergenic regions of the *Hoxb4* gene [40], which encodes a protein that promotes expansion of HSCs [42]. Furthermore, it is likely that a preferential hetero-dimerization of Pbx1 with Meis1 in HSCs influences the balance between their asymmetric and symmetric division, skewing it from asymmetric division to generate an equal number of HSCs and lineage-committed daughter cells toward symmetric self-renewal of HSCs, which is consistent with our finding that Prep1 deficiency increased cell cycling of HSPCs without causing their exhaustion. This notion gains supported from the observation that a homeodomain-less *C*. *elegans* orthologue of Prep/Meis, PSA3, is involved in asymmetric cell division, in concert with *C*. *elegans* orthologue of Pbx, CEH-20 [43]. In the hematopoietic system, NUP98-HOXA9, an oncogenic partner of Meis1 in human myeloid leukemia, has also been shown to promote symmetric self-renewal of hematopoietic precursors [44].

Our findings with adult HSPCs in the hematopoietic and endothelial cell-selective Prep1-CKO mice contrast with those in previous studies using *Prep1* hypomorphic embryos, which showed defective self-renewal activity of embryonic HSCs by using a serial transplantation assay into the irradiated host mice with competitor cells [45]. The exact reasons for this difference remain unclear; however, one possibility is that germline Prep1 insufficiency in *Prep1* hypomorphic mice extrinsically affects HSC functions through their niches in the fetal liver, because hematopoietic/endothelial cell-selective Prep1 deficiency had no drastic defect on the maintenance or differentiation of fetal HSPCs. Alternatively, the difference could be due to the hematopoietic conditions analyzed; steady-state hematopoiesis that we have examined in the Prep1-CKO mice and stress-hematopoiesis induced by serial transplantation of fetal liver HSCs with competitor cells in the previous studies [13, 45]. However, it is also conceivable that subtle differences in the genetic background of the *Prep1* hypomorphic mice generated from the 129/SvEvBrd-derived ES cells backcrossed onto C57BL/6 background for several generations [17, 20] versus the present mice generated directly from the C57BL/6-derived ES cells might affect the phenotypic consequences [46].

In addition to defects in adult BM HSPCs in Prep1-CKO mice, we also observed defective erythroid maturation and enhanced generation of the monocyte/macrophage-lineage cells, with apparently intact megakaryocytic- and granulocytic-lineage differentiation, conditions that have not been described in the BM of *Prep1*-hypomorphic mice. Although the cell numbers of MEP and GMP progenitor populations were elevated in CKO mice, these progenitor cells appear to have accumulated but not expanded, as BrdU incorporation by LK cells representing these progenitor populations was not altered in Prep1-CKO mice (Fig 3B). Since over-expression of Meis1 in CMPs induces lineage commitment towards the MEP fate [47], the absence of Prep1 might stabilize the function of Meis1 to promote lineage choice towards MEP. Nevertheless, it remains unclear how Prep1 impacts monocyte/macrophage development, e.g., by regulating the proliferation and survival of the mature monocytes and/or by modulating differentiation toward the monocyte lineage at the GMP stage of differentiation. A similar unresolved question is at which stage and how does Prep1 regulate erythroid- and megakaryocytic-lineage cell differentiation from MEP. One potential hint to explain the increase in the number of platelets is that both Mies1 and Prep1 bind the promoter region of the gene encoding platelet factor 4 (PF4), which is expressed from megakaryocyte to platelet stages [48, 49], so if there is a competition between Prep1 and Meis1 to occupy the *PF4* gene binding site, Prep1 might act as an inhibitor of platelet production from megakaryocytes.

One feature shared by the germline *Prep1*-hypomorphic and the hematopoietic and endothelial cell-selective Prep1-CKO mice is lymphoid cell differentiation, though detailed differences still exist. *Prep1*-hypomorphic mice show a reduction in CD4 SP and the CD8 SP thymocyte cell numbers, while preserving absolute cell numbers in both the DP and DN subsets, whereas in the Prep1-CKO mice the absolute cell numbers of DP thymocyte as well as SP thymocyte were significantly reduced. Conversely, while T cell development in the thymus was more severely affected in CKO mice than in *Prep1*-hypomorphic mice, mature T cell numbers in the spleen were significantly reduced in *Prep1*-hypomorphic but not in CKO mice. This may be due to compensation of peripheral T cell numbers by homeostatic expansion of the residual small population of peripheral T cells, which is promoted by the more severe defect in the output of naïve T cells from the thymus of CKO mice than *Prep1*-hypomorphic mice. In terms of B-lineage cell differentiation, we failed to detect the partial block in the transition from pro-B to pre-B cells in the adult BM of Prep1-CKO mice that has been reported by using tamoxifen-inducible *Rosa26-CreER*-mediated *Prep1* deletion [15], suggesting a non-B cell-autonomous function of Prep1 in the BM.

Overall the current work stringently confirms the previous findings using *Prep1* hypomorphic mice that Prep1 is required for erythroid and thymocyte differentiation, and further has uncovered a physiological role for Prep1 in HSPC expansion and myeloid differentiation. Further analysis of the impact of Prep1 deficiency on hematopoietic lineage-cell differentiation using appropriate lineage-specific *Cre* driver mice is required for a complete understanding of the function of Prep1 in the development of different hematopoietic lineage cells. Furthermore, comparison of the phenotypes caused by distinct mutant *Prep1* alleles will provide a clue to understand the homeodomain-dependent and-independent functions of Prep1 in the context of the mutually interactive regulatory network comprised of the TALE family homedomain transcription factors.

## Materials and Methods

### Ethics Statement

All animal experiments were carried out under the ethical guidance of Tokyo University of Science, and protocols were reviewed and approved by the Tokyo University of Science Animal Care and Use Committee.

### Mice and gene targeting

Details of the generation of mice carrying the floxed allele of the *Prep1* gene (*Prep1* ^*fl/fl*^) will be described elsewhere. In brief, the targeting vector containing a 0.9-kb genomic fragment immediately upstream of the *lox*P-flanked 1.2-kb fragment containing exon 3 of the *Prep1* gene and a 5.5-kb DNA fragment immediately downstream of the gene was electroporated into Bruce-4 C57BL/6-derived ES cells, and drug-resistant colonies were screened for homologous recombination. Targeted clones were injected into Balb/c blastocysts and the resultant chimeric mice were bred to produce progeny having germ line transmission of the mutated allele. F1 progeny harboring a targeted *Prep1* allele were then crossed with ubiquitous *CAG*-promoter-driven FLPe mice [50] on a C57BL/6 background to remove the FRT-flanked neomycin-resistant gene cassette. *Tie2-Cre* mice [26] as well as FLPe mice on a pure C57BL/6 background were provided by RIKEN Bioresource Center. The PCR primers for genotyping are listed in S1 Table.

### RT-PCR

Total RNA was isolated using Trizol reagent (Invitrogen) and first-strand cDNA was synthesized using oligo (dT) primers and a Superscript RT-PCR kit (Invitrogen). Each cDNA sample was then subjected to PCR. Amplified signals were confirmed to be single bands by gel electrophoresis. Specificity of products was confirmed by DNA sequencing. Primer sequences for PCR were listed in S1 Table.

### Western blotting

Cell lysates in NP40 buffer were separated by SDS-PAGE and transferred to PVDF membranes. The blots were then probed with an antibody raised against the N-terminal portion of Prep1 (ab55603, Abcam, Cambridge, MA, USA) and anti-GAPDH antibodies (Santa Cruz, CA), followed by HRP-conjugated secondary antibodies and then visualized by enhanced chemiluminescence (ECL plus). Blots were scanned and analyzed using a Luminescent image analyzer (LAS-3000, FUJIFILM, Japan).

### Peripheral blood count analysis

Blood samples were collected in EDTA-containing tubes and analyzed on a flow cytometer programmed with mouse-specific parameters (Sysmex, Hyogo, Japan).

### Flow cytometry

Single cell suspensions from the indicated organs were stained with a combination of FITC-, PE-,-APC,-APC-Cy7 and biotin-conjugated antibodies, followed by streptavidin-PerCP-Cy5.5 or streptavidin-PerCP-Cy7 (eBioscience, San Diego, CA). The conjugated and unconjugated antibodies specific to the following antigens were purchased from BD Biosciences (San Jose, CA) and eBioscience: TCRαβ, NK1.1 (PK136), CD3ε (145-2C11), CD11c (N418), CD135/flt3 (A2F10), B220 (RA3-6B2), c-Kit (2B8), CD4 (L3T4), CD8α (53–6.72), CD11b (M1/70), CD19 (1D3), TER119, Gr-1 (RB6-8C59), FcγI/III (2.4G2), Sca-1 (E13-161.7), IL-7Rα (A7R34), CD41 (MWReg30), CD48 (HM48-1), CD150 (TC15-12F12.2), CD43 (S7), and CD34 (RAM34). Dead cells were excluded using propidium iodide (PI) or 7-AAD. Gating schemes for hematopoietic stem/progenitor cells are shown in S4 Fig. Data were collected on FACSCantoII flow cytometers (BD Bioscience) and analyzed using FlowJo software (TreeStar, Ashland, OR). A FACSAreaII (BD Bioscience) was used for cell sorting.

### Cell cycle and proliferation analyses

For bromodeoxyuridine (BrdU) incorporation analysis, BrdU (BD Pharmingen) was injected intraperitoneally, 1 mg per mouse. At one hour post-injection, LSK and LK cells were collected from the BM, fixed, and stained with 7-AAD and anti-BrdU antibody using a FITC-BrdU Flow kit (BD Pharmingen), according to the manufacturer’s protocol. Pyronin Y (Sigma-Aldrich) and Hoechst 33342 (Sigma-Aldrich) staining was performed simultaneously within gated cells stained with CD150, CD48 and CD41.

### Statistical analysis

Statistical significance was calculated with the unpaired two-tailed Student’s *t*-test. Data were considered statistically significant when p values were less than 0.05.

## Supporting Information

S1 Fig Generation of a loss-of-function allele of *Prep1* in hematopoietic cells. (**A**) Diagram showing the targeting strategy for the mutant *Prep1* allele. (Top) *Prep1* exon 3 was flanked with *lox*P sites. Mating with appropriate Cre transgenic mice results in tissue-specific inactivation of *Prep1* due to Cre-mediated deletion of exon 3 (bottom). (**B**) Efficient tissue-specific deletion of *Prep1* in hematopoietic cells demonstrated by PCR performed on genomic DNA extracted from the BM of *Tie2-Cre; Prep1* ^*fl/fl*^ mice and *Prep1* ^*fl/fl*^ littermate controls using the primers P1 and P3 shown as small arrowheads in **A**. (**C**) PCR-amplified products of RNAs from the BM of *Tie2-Cre; Prep*1^*fl/fl*^ mice and *Prep1* ^*flfl*^ littermate controls using the primers corresponding to the exon 2 and exon 6 (P4 and P5, shown as small arrowheads in **A**). (**D**) cDNA and deduced amino acid sequences of the RT-PCR product from the BM cells of *Tie2-Cre; Prep1* ^*fl/fl*^ mice and *Prep1* ^*flfl*^ littermate controls. Primer pair used for RT-PCR was the same as in **C**. A frameshift in exon 4 was generated by excision of exon 3, disrupting the normal sequence encoded by exon 4 and generating a premature stop codon. (**E**) Western blot analysis of Prep1 protein expression in the BM of *Tie2-Cre; Prep1* ^*fl/fl*^ mice and *Prep1* ^*flfl*^ littermate controls. (TIF)
(TIF)

S2 Fig Prep1 expressed in hematopoietic/endothelial cells is dispensable for embryonic hematopoiesis in the fetal liver. **(A)** Representative flow cytometric profiles of hematopoietic progenitor cell populations (LSK; top panel, CLP; middle panel, CMP, GMP and MEP; bottom panel) from the fetal liver of *Tie2-Cre; Prep*1^*fl/fl*^ and *Prep1* ^*flfl*^ embryos (E 14.5). Numbers indicate percentage of gated cells among total fetal liver mononuclear cells. Bar graphs on the right depict absolute numbers of the indicated cell populations in total fetal liver mononuclear cells from *Tie2-Cre; Prep*1^*fl/fl*^ (solid bars) and control *Prep1* ^*flfl*^ (open bars) embryos (mean and SD; n = 4). (**B**) Representative flow cytometric profiles of lineage-committed cell populations in the fetal liver. Bar graphs on the right depict absolute numbers of the indicated cell populations in total fetal liver mononuclear cells from *Tie2-Cre; Prep*1^*fl/fl*^ (solid bars) and control *Prep1* ^*flfl*^ (open bars) embryos (mean and SD; n = 4). B-lineage cells (CD19^+^ Gr-1^-^), granulocytes (Gr-1^+^ CD11b^+^), monocytes (Gr-1^-^ CD11b^+^), proerythroblasts (I; Ter119^low^ CD71^high^), basophilic erythroblast (II; Ter119^high^ CD71^high^) and late erythroblasts (III; Ter119^high^ CD71^int^ and IV; Ter119^high^ CD71^low^). (TIF)
(TIF)

S3 Fig Differentiation of megakaryocytic-lineage cells in the bone marrow of *Tie2-Cre; Prep1* ^*fl/fl*^ mice. Representative flow cytometric profiles of megakaryocytic-lineage cell populations from *Tie2-Cre; Prep1* ^*fl/fl*^ mice and *Prep1* ^*fl/fl*^ littermate controls. Numbers indicate percentage of gated cells among the total cells analyzed; pro-megakaryocytes (c-Kit^+^ CD41^+^) and megakaryocytes (c-Kit^-^ CD41^+^). Bar graphs on the right depict absolute numbers of the indicated cell populations in the BM of two femurs from *Tie2-Cre; Prep*1^*fl/fl*^ (solid bars) and control *Prep1* ^*flfl*^ littermate (open bars) mice (mean and SD; n = 3). (TIF)
(TIF)

S4 Fig Flow cytometry gating strategy for hematopoietic stem/progenitor cells in the bone marrow. (**A**) Flow gating schemes for CD34^+/-^ and Flt3^+^ LSK and CLP in the BM of *Tie2-Cre; Prep1* ^*fl/fl*^ mice (CKO) and *Prep1* ^*fl/fl*^ littermates (control). Lineage markers (Lin) include CD11b, CD3, B220, Ter119, Gr-1 and 7-AAD. (B) Flow gating schemes for CMP, GMP and MEP in the BM of *Tie2-Cre; Prep1* ^*fl/fl*^ mice (CKO) and *Prep1* ^*fl/fl*^ littermates (control). Lineage markers (Lin) include CD11b, CD3, B220, Ter119, Gr-1, IL-7Rα and Sca-1. (**C**) Flow gating schemes for cell cycle analyses of CD150^+^ CD48^-^ CD41^-^ SP cells in the BM of *Tie2-Cre; Prep1* ^*fl/fl*^ mice (CKO) and *Prep1* ^*fl/fl*^ littermates (control). (TIF)
(TIF)

S1 Table List of primers for PCR (doc). (DOC)
