# Supplementary figures and images for: Loss of the Homeodomain Transcription Factor Prep1 Perturbs Adult Hematopoiesis in the Bone Marrow
Source: PLoS One. 2015 Aug 18;10(8):e0136107. doi: 10.1371/journal.pone.0136107 (PMC4540428; doi:10.1371/journal.pone.0136107)

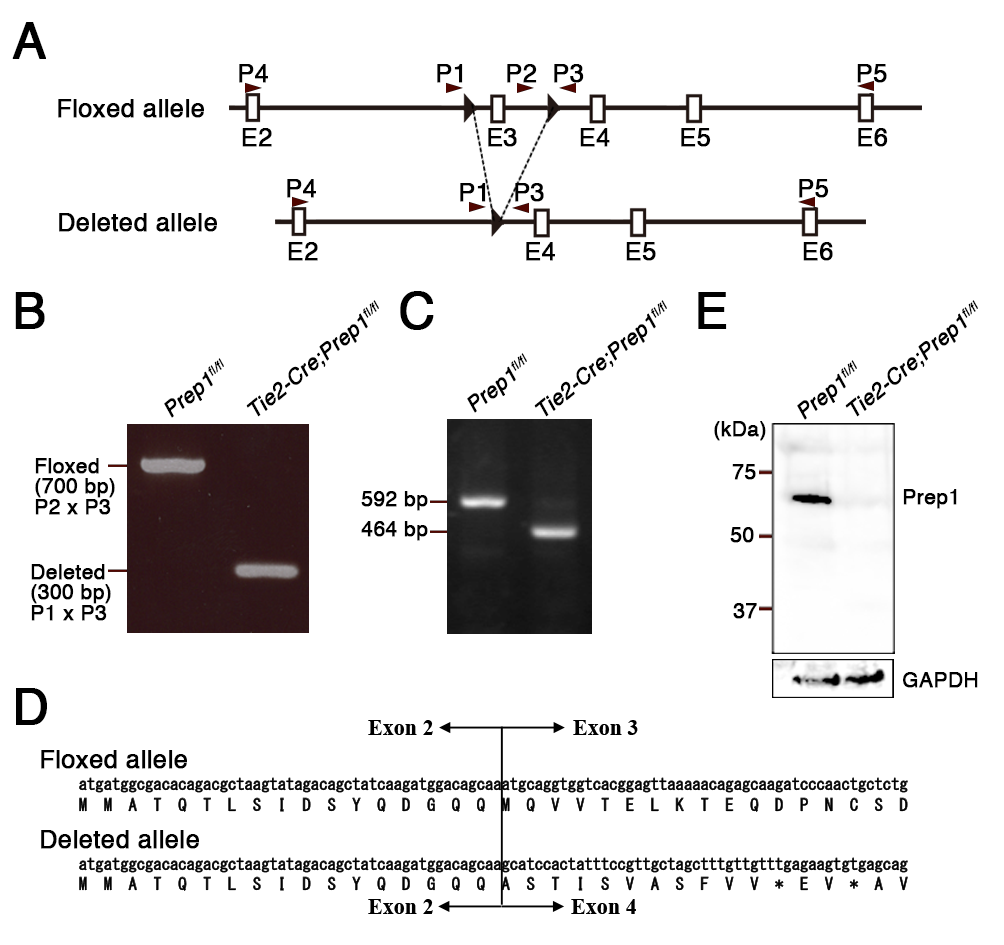

Supplement: S1 Fig — (A) Diagram showing the targeting strategy for the mutant Prep1 allele. (Top) Prep1 exon 3 was flanked with loxP sites. Mating with appropriate Cre transgenic mice results in tissue-specific inactivation of Prep1 due to Cre-mediated deletion of exon 3 (bottom). (B) Efficient tissue-specific deletion of Prep1 in hematopoietic cells demonstrated by PCR performed on genomic DNA extracted from the BM of Tie2-Cre; Prep1 fl/fl mice and Prep1 fl/fl littermate controls using the primers P1 and P3 shown as small arrowheads in A. (C) PCR-amplified products of RNAs from the BM of Tie2-Cre; Prep1fl/fl mice and Prep1 flfl littermate controls using the primers corresponding to the exon 2 and exon 6 (P4 and P5, shown as small arrowheads in A). (D) cDNA and deduced amino acid sequences of the RT-PCR product from the BM cells of Tie2-Cre; Prep1 fl/fl mice and Prep1 flfl littermate controls. Primer pair used for RT-PCR was the same as in C. A frameshift in exon 4 was generated by excision of exon 3, disrupting the normal sequence encoded by exon 4 and generating a premature stop codon. (E) Western blot analysis of Prep1 protein expression in the BM of Tie2-Cre; Prep1 fl/fl mice and Prep1 flfl littermate controls. (TIF) (TIF) [file pone.0136107.s001.tif]

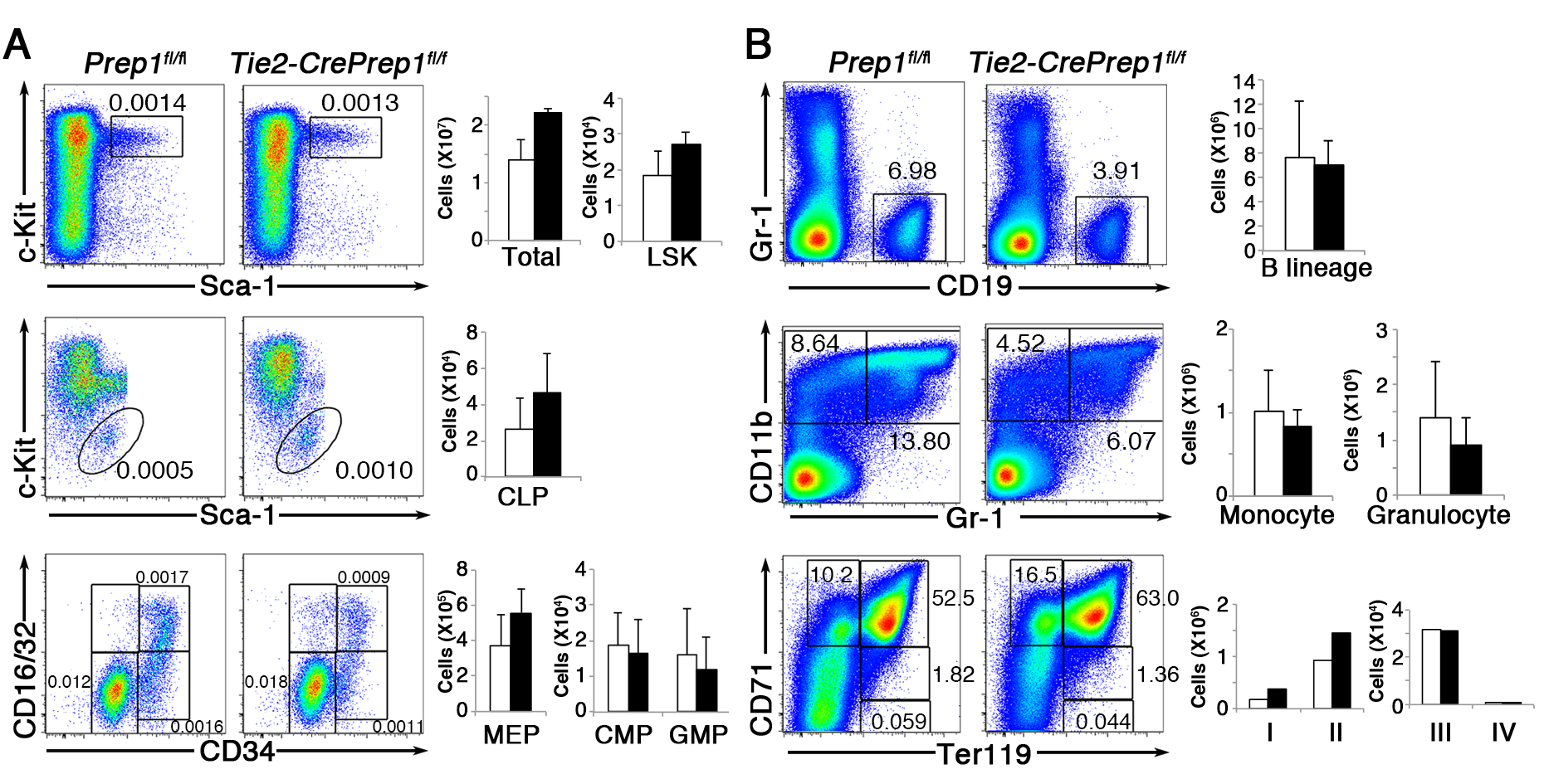

Supplement: S2 Fig — (A) Representative flow cytometric profiles of hematopoietic progenitor cell populations (LSK; top panel, CLP; middle panel, CMP, GMP and MEP; bottom panel) from the fetal liver of Tie2-Cre; Prep1fl/fl and Prep1 flfl embryos (E 14.5). Numbers indicate percentage of gated cells among total fetal liver mononuclear cells. Bar graphs on the right depict absolute numbers of the indicated cell populations in total fetal liver mononuclear cells from Tie2-Cre; Prep1fl/fl (solid bars) and control Prep1 flfl (open bars) embryos (mean and SD; n = 4). (B) Representative flow cytometric profiles of lineage-committed cell populations in the fetal liver. Bar graphs on the right depict absolute numbers of the indicated cell populations in total fetal liver mononuclear cells from Tie2-Cre; Prep1fl/fl (solid bars) and control Prep1 flfl (open bars) embryos (mean and SD; n = 4). B-lineage cells (CD19+ Gr-1-), granulocytes (Gr-1+ CD11b+), monocytes (Gr-1- CD11b+), proerythroblasts (I; Ter119low CD71high), basophilic erythroblast (II; Ter119high CD71high) and late erythroblasts (III; Ter119high CD71int and IV; Ter119high CD71low). (TIF) (TIF) [file pone.0136107.s002.tif]

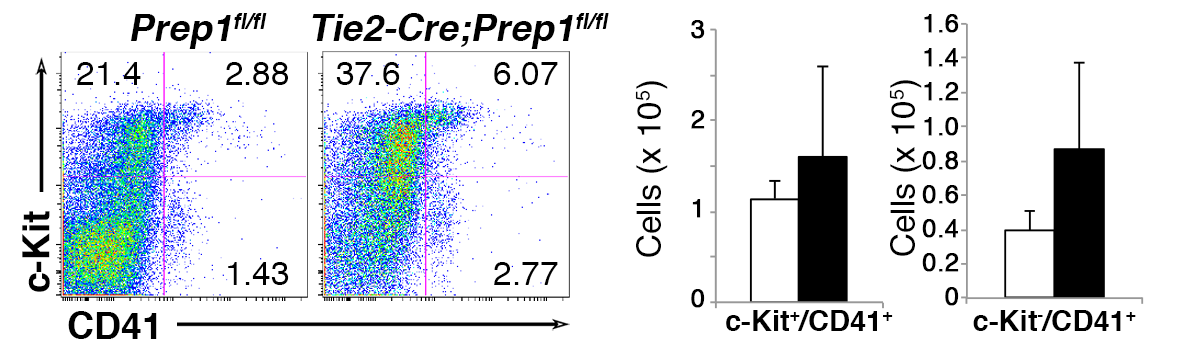

Supplement: S3 Fig — Representative flow cytometric profiles of megakaryocytic-lineage cell populations from Tie2-Cre; Prep1 fl/fl mice and Prep1 fl/fl littermate controls. Numbers indicate percentage of gated cells among the total cells analyzed; pro-megakaryocytes (c-Kit+ CD41+) and megakaryocytes (c-Kit- CD41+). Bar graphs on the right depict absolute numbers of the indicated cell populations in the BM of two femurs from Tie2-Cre; Prep1fl/fl (solid bars) and control Prep1 flfl littermate (open bars) mice (mean and SD; n = 3). (TIF) (TIF) [file pone.0136107.s003.tif]

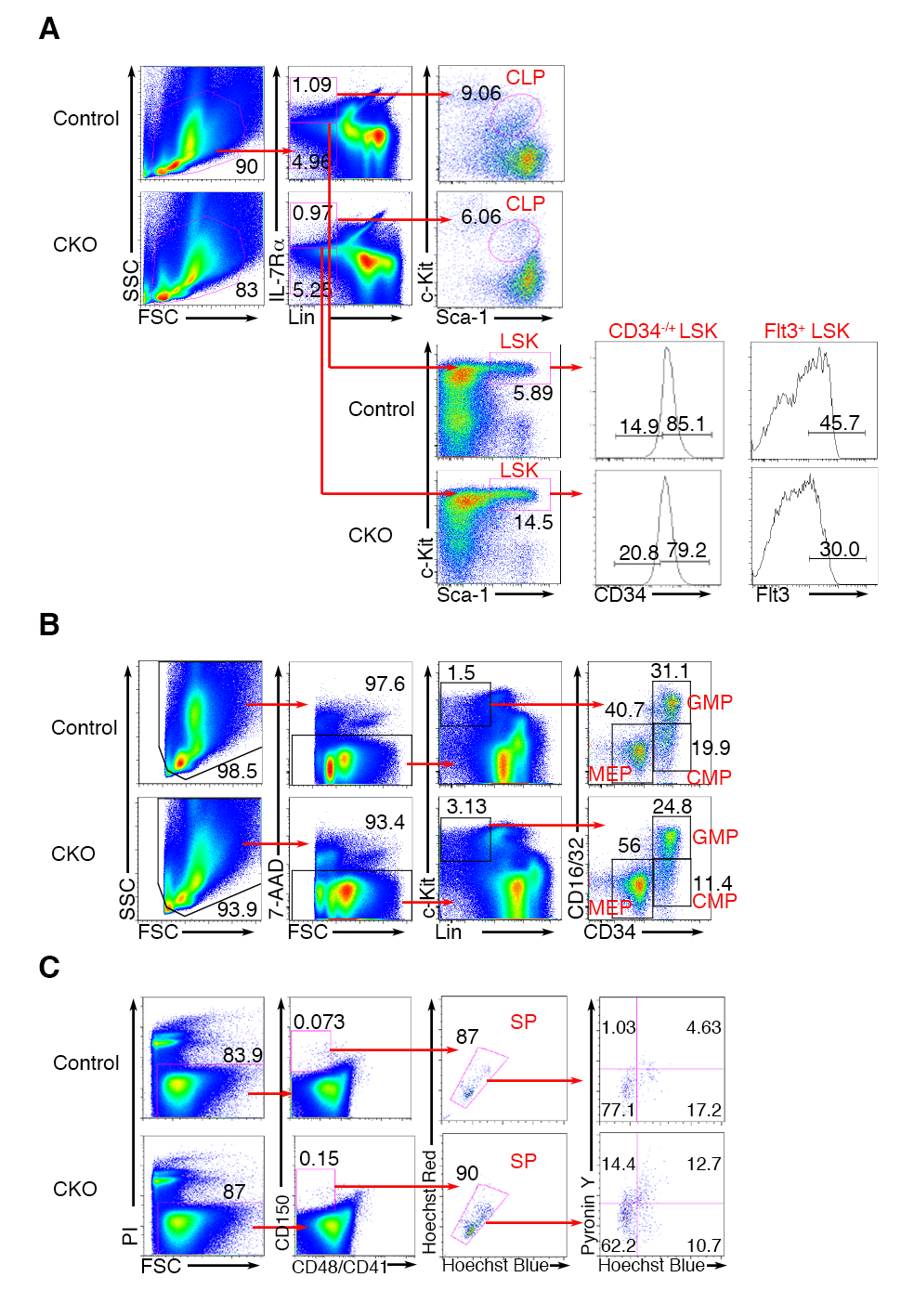

Supplement: S4 Fig — (A) Flow gating schemes for CD34+/- and Flt3+ LSK and CLP in the BM of Tie2-Cre; Prep1 fl/fl mice (CKO) and Prep1 fl/fl littermates (control). Lineage markers (Lin) include CD11b, CD3, B220, Ter119, Gr-1 and 7-AAD. (B) Flow gating schemes for CMP, GMP and MEP in the BM of Tie2-Cre; Prep1 fl/fl mice (CKO) and Prep1 fl/fl littermates (control). Lineage markers (Lin) include CD11b, CD3, B220, Ter119, Gr-1, IL-7Rα and Sca-1. (C) Flow gating schemes for cell cycle analyses of CD150+ CD48- CD41- SP cells in the BM of Tie2-Cre; Prep1 fl/fl mice (CKO) and Prep1 fl/fl littermates (control). (TIF) (TIF) [file pone.0136107.s004.tif]
