# Supplementary material for: Loss of the Homeodomain Transcription Factor Prep1 Perturbs Adult Hematopoiesis in the Bone Marrow
Source: PLoS One. 2015 Aug 18;10(8):e0136107. doi: 10.1371/journal.pone.0136107 (PMC4540428; doi:10.1371/journal.pone.0136107)
Supplement: S1 Table — (DOC) [file pone.0136107.s005.doc]

| S1 Table. Primer sequences used for genotyping and RT-PCR. | | |  |
| --- | --- | --- | --- |
|  |  |  |  |
| allele name |  | Primer sequence |  |
| **Prep1 deletion check** |  |  |  |
| Primer1 (P1) | F: | GACCTTCACATAGCTGCGCAGTTG |  |
| Primer2 (P2) | F: | GGATCCTAGTGAACCTCTTCGAGG |  |
| Primer3 (P3) | R: | CGCACAGGAAGATGAGTGACTGC |  |
|  |  |  |  |
| **Genotyping** |  |  |  |
| For seq SA | F: | TTACAACCTAGTAAACACAGCTGG |  |
| For seq int | R: | GAGGTTCTAGGAAGCAAACTTCAGC |  |
|  |  |  |  |
| Tie2-Cre | F: | CCCTGTGCTCAGACAGAAATGAGA |  |
|  | R: | CGCATAACCAGTGAAACAGCATTGC |  |
|  |  |  |  |
| TNAP-Cre | F: | CCAGAAGGTACCCCATTGTATGG |  |
|  | R: | GCATAACCAGTGAAACAGCAT |  |
|  |  |  |  |
| **RT-PCR** |  |  |  |
| Primer4 (P4) | F | GTGGACTCTCACTAACCATGATGGCG |  |
| Primer5 (P5) | R | GGCACCACGATTCCCTGGGGGCTGAGC | |
|  |  |  |  |
| F; Forward, R; Reverse |  |  |  |
